# Six New Triterpene Derivatives from *Aralia chinensis* Var. *dasyphylloides*

**DOI:** 10.3390/molecules21121700

**Published:** 2016-12-09

**Authors:** Rui-xi Gao, Maochuan Liao, Xianju Huang, Yu Chen, Guangzhong Yang, Jun Li

**Affiliations:** 1 Laboratory for Natural Product Chemistry, College of Pharmacy, South-Central University for Nationalities, Wuhan 430074, China; gaoruixi@gmail.com; 2 College of Pharmacy, South-Central University for Nationalities, Wuhan 430074, China; sippr1976@163.com (M.L.); xianju@mail.scuec.edu.cn (X.H.); yanggz888@126.com (G.Y.); 3 College of Chemistry and Material Sciences, South-Central University for Nationalities, Wuhan 430074, China; chenyuwh888@126.com

**Keywords:** *Aralia chinensis* var. *dasyphylloides*, triterpene derivatives, araliachinolic acids I and II, araliachinosides I–IV

## Abstract

*Aralia chinensis* var. *dasyphylloides* is widely distributed in China and used as a traditional herbal medicine for the treatment of digestive and immune system diseases. The present study aimed to search for novel oleanolic-type triterpenoids in low-polarity fractions. Six new triterpene derivatives (**1**–**6**), together with two known compounds were isolated from the barks of *A. chinensis* var. *dasyphylloides*. Their structures were elucidated by 1D- and 2D-NMR spectroscopic analysis and chemical methods. They were identified as 3-oxo-oleana-11,13(18)-dien-28,30-dioic acid (**1**), 30-hydroxy-3-oxo-oleana-11,13(18)-dien-28-oic acid (**2**), 3β-hydroxy-oleana-11,13(18)-dien-28-oic acid-28-*O*-β-d-glucopyranoside (**3**), 3β,30-dihydroxy-oleana-11,13(18)-dien-28-oic acid-28-*O*-β-d-glucopyranoside (**4**), 3β-hydroxy-oleana-11,13(18)-dien-28-oic acid-3-*O-*β-d-xylopyranosyl-(1 → 2)-β-d-glucopyranoside (**5**), 3β,29-dihydroxy-oleana-9(11),12-dien-28-oic acid-28-*O*-β-d-glucopyranoside (**6**), namely, araliachinolic acids I and II and araliachinosides I–IV. The cytotoxicity of the isolated compounds was tested against HepG2, A549, SGC7901, and MCF7 cell lines, but no apparent activity was observed at a concentration of 50 μM.

## 1. Introduction

*Aralia chinensis* Linn. var. *dasyphylloides* Hand.-Mazz. Symb. (Araliaceae) is distributed in the Sichuan, Guizhou, Guangxi, and Hubei provinces of China [1]. It has been used as a traditional herbal medicine for the treatment of gastric ulcer, hepatitis rheumatic arthritis, and other diseases. Previous phytochemical investigations on this plant revealed the presence of essential oil [2] and oleanolic-type triterpenoid saponins [3,4]. Those saponins demonstrated inhibitory activities against α-glucosidase [5], moderate antioxidant effects and antiglycation activities [6], and cytotoxic activities against human nasopharyngeal carcinoma epithelial (CNE) cells [7]. 

**Figure 1:**
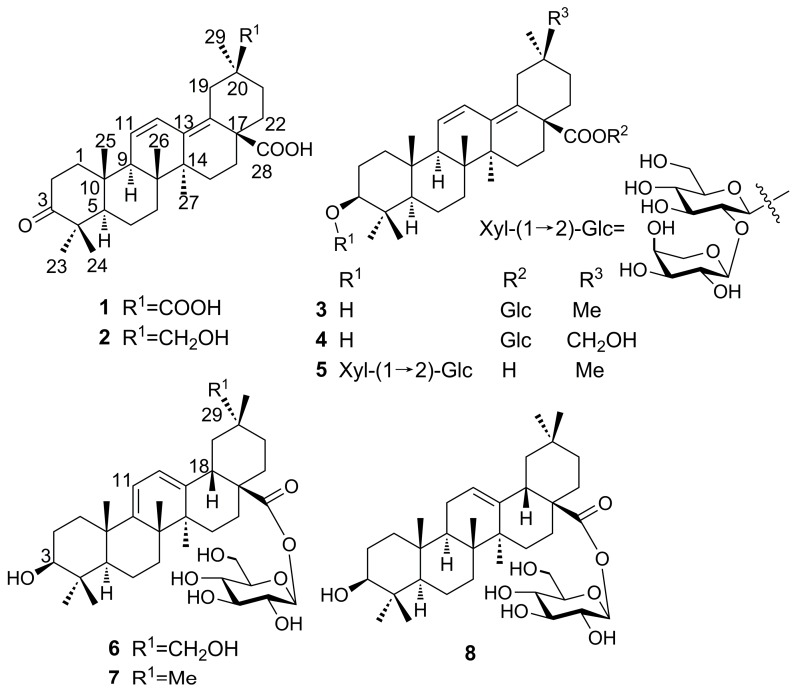
Chemical structures of triterpene derivatives **1**–**8.**

As part of our effort to search for novel oleanolic-type triterpenoids from *A. chinensis* var. *dasyphylloides*, we report here the isolation and structure determination of the new terpenoids **1**–**6** (Figure 1), together with two known saponins, oleana-9(11),12-diene-28-oic acid-28-*O*-β-d-glucopyranoside (**7**) [8] and oleanolic acid 28-*O*-β-d-glucopyranoside (**8**) [9] from the CHCl_3_ and EtOAc-soluble fractions.

## 2. Results and Discussion

The EtOH extract of the barks of *A. chinensis* var. *dasyphylloides* was fractionated by repeated medium-pressure liquid chromatography (MPLC) on normal and reversed-phase (RP) silica gel to yield the new derivatives **1**–**6** and two previously reported saponins (**7** and **8**). The structures of the new compounds were elucidated on the basis of extensive NMR spectroscopic analysis, including a series of 2D-NMR experiments (HSQC, HMBC, and NOESY), and mass spectrometry data. The known saponins (**7** and **8**) were identified by comparison of their spectral data with literature data [8,9].

**Figure 2:**
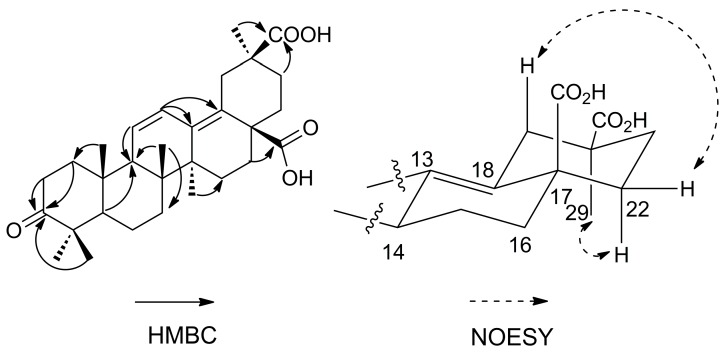
Key HMBC (Heteronuclear Multiple Bond Correlation, From H to C) and NOESY (Nuclear Overhauser Effect Spectroscopy, From H to H) correlations for **1.**

Compound **1** was obtained as a white amorphous powder with the molecular formula determined to be C_30_H_42_O_5_ on the basis of the molecular ion peak [M]^+^ at *m/z* 482.3033 (calcd. 482.3032) observed in its HR-EI-MS and NMR spectroscopic data. The ^1^H-NMR spectrum revealed six methyl group signals at δ (ppm) 1.09 (3H, s, Me-23), 1.04 (3H, s, Me-24), 1.04 (3H, s, Me-25), 0.86 (3H, s, Me-26), 0.93 (3H, s, Me-27), and 1.17 (3H, s, Me-29), as well as two *cis* olefinic protons at δ (ppm) 5.64 (1H, d, *J =* 11.0 Hz) and 6.73 (1H, dd, *J =* 11.0, 3.0 Hz). The ^13^C-NMR and DEPT spectra revealed six methyls, nine methylenes, two *sp*^3^ methines at δ (ppm) 55.6 (C-5) and 55.1 C-9), six *sp*^3^ quaternary carbon signals, four olefinic signals at δ (ppm) 127.8 (C-11), 126.6 (C-12), 137.1 (C-13), and 133.7 (C-18), and three carbonyl signals at δ (ppm) 220.3 (C-3), 180.2 (C-28), and 181.9 (C-30). The ^13^C-NMR signals, especially signals at δ (ppm) 55.6 C-5), 55.1 (C-9), 127.8 (C-11), 126.6 (C-12), 137.1 (C-13), and 133.7 (C-18), indicated the presence of an oleana-11,13(18)-diene-type triterpene, as confirmed by comparison with 3-oxo-11,13(18)-oleanadien-28-oic acid [10]. The main difference was the carboxyl group at C-30 and the down-field shift of C-20 at δ 45.7 ppm in **1**. The carboxyl group also caused the downfield shift of C-29 at δ 29.0 ppm, as compared to δ 24.0 ppm in compounds with a C-30 methyl group [10,11,12]. In the HMBC spectrum (Figure 2), the correlation between the Me-24 protons at δ 1.04 ppm and C-3 (δ 220.3 ppm) revealed that one carbonyl group was located at C-3. The position of the second carboxyl group was determined by the HMBC correlation between Me-29 protons at δ 1.17 ppm and C-30 (δ 181.9 ppm), which could be confirmed by the NOESY correlations of H_β_-19 (δ 3.29 ppm) with H_β_-22 (δ 2.30 ppm), H_α_-22 (δ 1.34 ppm) with Me-29 (δ 1.17 ppm). The position of the third carboxyl group was determined by the HMBC correlation between H-16 at δ 1.96 ppm and C-28 (δ 180.2 ppm). Very recently, a compound has been reported as 3-oxooleana-11,13(18)-diene-28,30 dioic acid [13]. However, in this reference, the structure depicted corresponds to the 29-carboxylic acid derivative, and no evidence is provided for the location of the carboxylic group at C-29 or C-30. Thus, the structure of compound **1** is assigned here unambiguously for the first time (The NMR data was available at the Supplementary Materials), and the compound was named araliachinolic acid I.

Compound **2** was obtained as a white amorphous powder with the molecular formula determined to be C_30_H_44_O_4_ from the pseudo-molecular ion peak [M − H]^−^ at *m*/*z* 467.3159 (calcd. 467.3161) observed in its HR-ESI-MS and its NMR spectroscopic data. The ^1^H- and ^13^C-NMR data (Table 1) of **2** were similar to those of **1**. Careful comparison of the NMR data between compound **1** and **2** indicated that both compounds possessed the same carbon skeleton, but had different substitution at C-30. Compared with compound **1**, the ^1^H-NMR spectrum of **2** showed a pair of doublets at δ (ppm) 3.18 (1H, d, *J =* 10.0 Hz) and 3.38 (1H, t, *J =* 10.0 Hz), which were assigned to CH_2_-30. In the ^13^C-NMR spectrum, C-30 was observed at δ 67.0 ppm and C-20 was downfield shifted at δ 37.5 ppm. In the HMBC spectrum, the correlation between Me-29 protons at δ 0.90 ppm and C-30 (δ 67.0 ppm) revealed the hydroxymethylene group to be located at C-30. The carbonyl group was positioned at C-3 based on the correlation between the Me-23 protons at δ 1.07 ppm and C-3 (δ 220.3 ppm). The diene structure was confirmed by the correlations between H-11 and H-12 protons (δ 6.50 ppm and 5.64 ppm) and C-13 (δ 138.0 ppm) and C-18 (δ 133.8 ppm), respectively. The position of the second carboxyl group was determined by the correlation between H-22 (δ 2.19 ppm) and C-28 (δ 180.2 ppm). Thus, compound **2** was a new compound, named araliachinolic acid II. 

Compound **3** was obtained as a white amorphous powder with the molecular formula determined to be C_36_H_56_O_8_ from the pseudo-molecular ion peak [M − H]^−^ at *m*/*z* 615.3895 (calcd. 615.3897) evident in its HR-ESI-MS and its NMR spectroscopic data. The NMR data of **3** (Table 1) were similar to those of 3β-hydroxy-11,13(18)-oleanedien-28-oic acid [14]. The main differences in the NMR data of these two compounds were the signals of a sugar moiety in the case of **3**. Thus, in the ^1^H-NMR spectrum of **3**, the anomeric signal at δ 5.45 ppm (1H, d, *J =* 8.5 Hz) and further signals at δ (ppm) 3.27, 3.37, 3.30, 3.31, 3.66, and 3.85 revealed the presence of one sugar which could be identified as β-d-glucopyranoside by acid hydrolysis, derivatization, and HPLC analysis. In the HMBC spectrum, the β-d-glucose was linked to the carboxyl group at C-28, based on the up-field shift from δ 180.2 ppm to δ 176.9 ppm. The correlation between Me-24 protons at δ 0.77 ppm and C-3 (δ 79.7 ppm) confirmed the presence of one hydroxyl group at C-3. This hydroxyl group was β-oriented by the NOESY correlation of H-3 (δ 3.18 ppm) with H-5 (δ 0.83 ppm) (The NOESY data was available at the Supplementary Materials). Taken together, these data indicate compound **3** to be a new compound, which was named araliachinoside I. 

Compound **4** was obtained as a white amorphous powder with the molecular formula determined to be C_36_H_56_O_9_ on the basis of the pseudo-molecular ion peak [M + Cl]^−^ at *m*/*z* 667.3616 (calcd. 667.3613) observed in its HR-ESI-MS and its NMR spectroscopic data. A comparison of the NMR data between **4** and **3** indicated that the two compounds possessed the same structure, with the only difference being the substitution of the methyl group at C-30 in **3** by a hydroxymethylene group in compound **4**. This was confirmed by the correlation between Me-29 protons at δ 1.17 ppm and C-30 (δ 66.4 ppm) in the HMBC spectrum. β-d-Glucose was identified via HPLC analysis after acid hydrolysis and derivatization. These data indicated that compound **4** was a new compound, which was named as araliachinoside II. 

Compound **5** was obtained as a white amorphous powder with the molecular formula determined to be C_41_H_64_O_12_ from the pseudo-molecular ion peak [M + Cl]^−^ at *m*/*z* 783.4086 (calcd. 783.4086) evident in its HR-ESI-MS and its NMR spectroscopic data. A comparison of the NMR data of **5** with 3β-hydroxy-oleane-11,13(18)-dien-28-oic acid-3-*O*-β-d-glucopyranosyl-(1 → 2)-β-d-xylopyranoside [7] showed that the two compounds were almost identical. After careful comparison, the sequence of the two monosaccharides in the two compounds was shown to be different. In the HMBC spectrum of compound **5**, the anomeric signal at δ 4.98 ppm (β-d-glucose) was correlated with C-3 of the aglycon moiety (δ 89.3 ppm), which indicated the β-d-glucose to be directly linked to the aglycon. The interglycosidic linkage was established based on the correlation of the anomeric signal at δ 5.32 ppm (β-d-xylose) with C-2 of β-d-glucose (δ 84.2 ppm). Taken together, these data indicated compound **5** to be a new compound, named araliachinoside III. 

**Figure 3:**
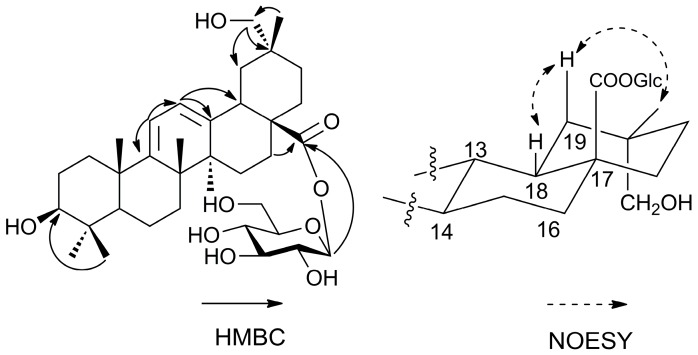
Key HMBC (From H to C) and NOESY (From H to H) correlations for **6.**

Compound **6** was obtained as a white amorphous powder with the molecular formula determined to be C_36_H_56_O_9_ from the pseudo-molecular ion peak [M − H]^−^ at *m*/*z* 631.3847 (calcd. 631.3846) observed in its HR-ESI-MS and its NMR spectroscopic data. The ^1^H-NMR data (Table 1) of the aglycon moiety in **6** revealed six methyl groups at δ (ppm) 1.25 (3H, s, Me-23), 1.07 (3H, s, Me-24), 1.24 (3H, s, Me-25), 1.47 (3H, s, Me-26), 1.22 (3H, s, Me-27), and 1.09 (3H, s, Me-30), two methine signals at δ (ppm) 0.99 (1H, s, H-5) and 3.51 (1H, m, H-18), and two olefinic signals at δ (ppm) 5.76 (1H, d, *J =* 5.6 Hz) and 5.80 (1H, d, *J =* 5.6 Hz). In addition, the anomeric signal observed at δ 6.41 ppm (1H, d, *J =* 8.0 Hz), with further signals at δ (ppm) 4.25, 4.29, 4.40, 4.04, 4.42, and 4.48, revealed that compound **6** contained a β-d-glucose moiety, which was confirmed after acid hydrolysis, derivatization, and HPLC analysis. The ^13^C-NMR and DEPT spectra showed six methyls, nine methylenes, two *sp*^3^ methines at δ (ppm) 51.7 (C-5) and 39.8 (C-18), six quaternary *sp*^3^ carbons, one oxygenated methine (δ 77.8 ppm, C-3), one carboxyl group (δ 176.8 ppm, C-28), and one hydroxymethylene group (δ 73.6 ppm, C-29). Moreover, four olefinic signals were observed at δ (ppm) 155.8 (C-9), 116.1 (C-11), 121.3 (C-12), and 145.9 (C-13), as well as a group of β-d-glucose signals at δ (ppm) 96.0 (glc-1), 74.2 (glc-2), 78.9 (glc-3), 71.0 (glc-4), 79.4 (glc-5), and 62.4 (glc-6). Taken together, these signals were similar to those of oleana-9(11), 12-diene-28-oic acid-28-*O*-β-d-glucopyranoside (**7**) [8]. A detailed comparison of the HMBC data between **6** (Figure 3) and **7** revealed that the main difference was the presence of a hydroxymethylene group at C-29. This was in particular revealed by the correlation between Me-30 protons (δ 1.09 ppm) and C-29 (δ 73.6 ppm). The NOESY correlations (Figure 3) of H-18 (δ 3.51 ppm) with H_β_-19 (δ 2.12 ppm) and Me-30 (δ 1.09 ppm) confirmed the position of the hydroxymethylene group at C-29. Thus, the structure of compound of **6** was identified as shown in Figure 1 and named as araliachinoside IV. 

Since triterpene saponins with an acyl group have been reported to show selective cytotoxic activities [7], the cytotoxicity of compounds **2** and **4**–**8** was tested against HepG2, A549, SGC7901, and MCF7 cell lines. However, none of these compound showed any apparent cytotoxicity (IC_50_ > 50 μM). 

## 3. Materials and Methods

### 3.1. General

Column chromatography (CC) was performed using silica gel (200–300 mesh, 300–400 mesh, Qingdao Haiyang Chemical Group Co., Qingdao, China). Thin-layer chromatography was performed on silica gel GF254 (Qingdao Haiyang Chemical Group Co., Qingdao, China). MCI was purchased from Mitsubishi Chemical Group Co. (Tokyo, Japan) Semi-preparative HPLC was performed on a DIONEX Ultimate 3000 system equipped with a diode array detector and a C18 column (250 mm × 10 mm, 5 μm, YMC Co. Ltd., Kyoto, Japan). HR-EI-MS was measured on a Waters Autospec Premier 776 mass spectrometer (Waters, Milford, MA, USA). HR-ESI-MS was recorded on an Agilent G6230 TOF mass spectrometer (Agilent Technologies, Santa Clara, CA, USA). NMR spectra were obtained on a Bruker DMX-500 spectrometer (Bruker, Karlsruher, Germany) using TMS as an internal reference. l-cysteine methyl ester and standard monosaccharides (d-glucose and d-xylose) used in HPLC experiments were purchased from Aladdin industrial Co. Ltd. (Shanghai, China). *O*-Tolyl-isothiocyanate was obtained from Sigma-Aldrich Co. Ltd (Sigma-Aldrich China, Shanghai, China). Other chemical reagents were purchased from Sinopharm Chemical Reagent Co. Ltd. Shanghai, China.

### 3.2. Plant Material

The barks of *A. chinensis* var. *dasyphylloides* were collected in June 2016 from Li Chuan City, Hubei Province, China. They were identified by Dr. Xinqiao Liu from College of Pharmacy at South-Central University for Nationalities, China. A voucher specimen (No. EP-201606) was deposited at the herbarium of College of Pharmacy, South-Central University for Nationalities, China.

### 3.3. Extraction and Isolation

The dried and powdered barks (4.5 kg) of *A. chinensis* var. *dasyphylloides* were extracted three times with 95% ethanol at room temperature (25 L, each 4 h). After removal of the solvent under reduced pressure, the ethanol extract was successively partitioned into petroleum ether (PE), CHCl_3_, EtOAc, and *n*-BuOH fractions. The CHCl_3_ soluble fraction (50 g) was subjected to CC (12 × 40 cm) over silica gel (200–300 mesh) and eluted with a gradient of CH_2_Cl_2_–MeOH (9:1, 8:2, 7:3, *v/v*) to yield 3 fractions (Fractions 1–3). Fraction 2 (5 g) was subjected to a CC (6 × 45 cm) over silica gel (300–400 mesh) with cyclohexane–acetone (9:1, 8:2, 7:3, 0:1, *v/v*) to yield 4 subfractions (Fractions 2.1–2.4). Fraction 2.3 (2 g) was subjected to CC (2 × 50 cm) over silica gel (300–400 mesh) with cyclohexane–acetone (6:4, *v/v*) to yield 5 subfractions (Fractions 2.3.1–2.3.5). Fraction 2.3.2 (200 mg) was purified by semi-preparative HPLC using MeCN–H_2_O (55:45, *v/v*, 254 nm) to provide compounds **1** (10.2 mg), **2** (20.3 mg), **3** (5.2 mg), **7** (5.1 mg), and **8** (5.6 mg). The EtOAc soluble fraction (50 g) was subjected to CC (12 × 40 cm) over silica gel (200–300 mesh) and eluted with a gradient of CH_2_Cl_2_–MeOH (9:1, 8:2, 7:3, 6:4, 0:1, *v/v*) to yield 5 fractions (Fractions 01–05). Fraction 02 (8 g) was subjected to CC (6 × 55 cm) over silica gel (300–400 mesh) and eluted with a gradient of CH_2_Cl_2_–MeOH–H_2_O (9:1:0.1, 8:2:0.2, *v/v*) to yield 3 fractions (Fractions 02.1–02.3). Fractions 02.1 and 02.2 were combined into groups (marked as Fraction II, 2 g) based on their TLC patterns. Fraction II was subjected to MCI with MeOH–H_2_O (3:7, 4:6, *v/v*) to yield 4 subfractions (Fractions II.1–II.4). Fraction II.1 (1 g) was subjected to CC (1 × 50 cm) over silica gel (300–400 mesh) and eluted with a gradient of CH_2_Cl_2_–MeOH (9:1, 8:2, *v/v*) to yield 2 subfractions (Fractions II.1.1–II.1.2). Fraction II.1.1 (200 mg) and Fraction II.1.2 (100 mg) were purified by semi-preparative HPLC using MeCN–H_2_O (40:60 → 65:35, *v/v*, 40 min, 254 nm) to yield compounds **4** (19.8 mg), **5** (18.5 mg, from Fraction II.1.2), **6** (16.3 mg), and **8** (15.2 mg).

*Araliachinolic acid I* (**1**): White amorphous powder. [α]${}_{D}^{25}$ = −75.3° (*c* = 0.45, MeOH), HR-EI-MS: *m*/*z* 482.3033 [M]^+^ (calcd for C_30_H_42_O_5_, 482.3032), EI-MS: *m*/*z* 482 [M]^+^ (100), 483 [M + 1]^+^ (40), 437 [M − CO_2_H]^+^ (30), 315 (15), 285 (13), 245 (16), 219 (25), 173 (43). ^1^H-NMR (CD_3_OD, 500 MHz) and ^13^C-NMR (CD_3_OD, 125 MHz) (see Table 1).

*Araliachinolic acid II* (**2**): White amorphous powder. [α]${}_{D}^{25}$ = −85.1° (*c* = 0.35, MeOH), HR-ESI-MS: *m*/*z* 467.3159 [M − H]^−^ (calcd for C_30_H_43_O_4_, 467.3161), ESI-MS: *m*/*z* 467 [M − H]^−^, ^1^H-NMR (CD_3_OD, 500 MHz) and ^13^C-NMR (CD_3_OD, 125 MHz) (see Table 1).

*Araliachinoside I* (**3**): White amorphous powder. [α]${}_{D}^{25}$ = −71.5° (*c* =0.18, MeOH), HR-ESI-MS: *m*/*z* 615.3895 [M − H]^−^ (calcd for C_36_H_55_O_8_, 615.3897), ESI-MS: *m*/*z* 615 [M − H]^−^, ^1^H-NMR (CD_3_OD, 500 MHz) and ^13^C-NMR (CD_3_OD, 125 MHz) (see Table 1).

*Araliachinoside II* (**4**): White amorphous powder. [α]${}_{D}^{25}$ = −90.2° (*c* = 0.31, C_5_H_5_N), HR-ESI-MS: *m*/*z* 667.3616 [M + Cl]^−^ (calcd for C_36_H_56_O_9_Cl, 667.3613), ESI-MS: *m*/*z* 667 [M + Cl]^−^. ^1^H- NMR (C_5_D_5_N, 500 MHz) and ^13^C-NMR (C_5_D_5_N, 125 MHz) (see Table 1).

*Araliachinoside III* (**5**): White amorphous powder. [α]${}_{D}^{25}$ = −126.5° (*c* = 0.22, C_5_H_5_N), HR-ESI-MS: *m*/*z* 783.4086 [M + Cl]^−^ (calcd for C_41_H_64_O_12_Cl, 783.4086), ESI-MS: *m/z* 783 [M + Cl]^−^. ^1^H-NMR (C_5_D_5_N, 500 MHz) and ^13^C-NMR (C_5_D_5_N, 125 MHz) (see Table 1).

*Araliachinoside IV* (**6**): White amorphous powder. [α]${}_{D}^{25}$ = +122.1° (*c* = 0.15, C_5_H_5_N), HR-ESI-MS: *m*/*z* 631.3847 [M − H]^−^ (calcd for C_36_H_55_O_9_, 631.3846), ESI-MS: *m*/*z* 631 [M − H]^−^, ^1^H-NMR (C_5_D_5_N, 500 MHz) and ^13^C-NMR (C_5_D_5_N, 125 MHz) (see Table 1).

### 3.4. Acid Hydrolysis and Derivatization of ***3**–**6***

Each compound (2.5 mg) was hydrolyzed with 4N aqueous trifluoroacetic acid (TFA, 5 mL) for 3.5 h at 95 °C in a water bath. The mixture was diluted with water (10 mL), extracted with CH_2_Cl_2_ (three times, 5 mL each), and evaporated under reduced pressure to remove TFA. l-cysteine methyl ester hydrochloride (2.5 mg) was dissolved in anhydrous pyridine (1.0 mL) and added to the sugar residue. The solution was refluxed at 60 °C in the water bath for 2.5 h. *O*-Tolyl-isothiocyanate (10 μL) was added to the refluxed solution and heated for another 1 h. The reaction mixture was analyzed by HPLC on an Agilent HC-C18, 250.0 × 4.6 mm, 5 μm column at 30 °C with an isocratic elution of CH_3_CN–H_2_O (25:75, *v/v*, containing 1‰ TFA). The flow was 1.0 mL/min and detection was at 250 nm. The retention times of standard monosaccharides derivatized using the same procedure were 14.3 min (d-glucose) and 16.2 min (d-xylose). Comparison of the retention times of standards and samples enabled to establish the absolute configuration of monosaccharides in each hydrolysate.

### 3.5. MTT Assay for Measuring Cell Viability

The cell lines (HepG2, A549, SGC7901, and MCF7) were purchased from the cell bank of Chinese Academy of Sciences (Shanghai, China) and seeded in 96-well plates, incubated for 24 h. After incubation, cells were treated with compounds (50 μM) at 37 °C in 5% CO_2_ for 24 h. 10 μL of MTT (5 mg/mL, dissolved in DMEM) was added to each well, followed by incubation for 2–4 h. The medium was aspirated and formazan crystals were dissolved with 100 μL of DMSO. Optical density at 492 nm was determined with a microplate reader. Cells viability in response to treatment was calculated as percentage of control cells treated with DMSO. 

## Figures and Tables

**Table 1:** ^1^H- and ^13^C-NMR data (500 and 125 MHz, respectively, **1**–**3** in CD_3_OD, **4**–**6** in C_5_D_5_N) of **1**–**6**.

Position	1		2		3		4		5		6	
	δ_H_	δ_C_	δ_H_	δ_C_	δ_H_	δ_C_	δ_H_	δ_C_	δ_H_	δ_C_	δ_H_	δ_C_
1_α_	1.53 m	39.8	1.55 m	39.8	1.06 m	39.3	1.06 m	38.3	0.94 m	38.1	1.48 m	37.8
1_β_	2.15 m		2.15 m		1.92 m		1.86 m		1.75 m		2.02 m	
2_α_	2.49 m	34.8	2.49 m	34.8	1.64 m	26.0	1.91 br s	28.2	1.92 m	26.5	1.94 br s	29.0
2_β_	2.61 m		2.61 m		1.68 m				2.31 m			
3		220.3		220.3	3.18 m	79.7	3.48 m	77.9	3.35 m	89.3	3.46 m	77.8
4		47.8		47.8		40.0		39.6		40.0		39.8
5	1.49 m	55.6	1.49 m	55.6	0.83 s	56.3	0.87 s	55.1	0.79 s	55.3	0.99 s	51.7
6	1.39 m, 1.57 m	20.7	1.40 m, 1.56 m	20.7	1.48 m, 1.62 m	19.2	1.37 m, 1.56 m	18.6	1.40 m, 158 m	18.4	1.46 m, 1.60 m	18.8
7	1.38 m	32.8	1.40 m	32.8	1.35 br s	32.6	1.25 s	32.4	1.31 s	33.1	1.77 m, 1.45 m	32.6
8		41.6		41.8		42.0		41.3		41.2		41.5
9	2.08 s	55.1	2.08 s	55.0	1.98 s	55.8	2.01 s	54.6	2.00 s	54.7		155.8
10		37.9		37.9		38.3		37.7		36.9		39.5
11	6.73 dd (11.0, 3.0)	127.8	6.50 dd (10.5, 3.0)	127.6	6.45 dd (10.5, 2.5)	126.5	6.85 dd (10.5, 2.0)	126.1	6.67 dd (10.5, 2.0)	126.1	5.76 d (5.5)	116.1
12	5.64 d (11.0)	126.6	5.64 d (10.5)	126.9	5.67 d (10.5)	128.0	5.75 d (10.5)	127.4	5.73 d (10.5)	126.7	5.80 d (5.5)	121.3
13		137.1		138.0		138.3		137.4		137.0		145.9
14		43.3		43.3		43.2		42.1		42.7		43.3
15_α_	1.05 m	26.2	1.05 m	26.2	1.03 m	24.6	1.02 m	25.2	1.83 m	25.9	1.25 m	27.5
15_β_	1.68 m		1.68 m		1.74 m		2.10 m		2.30 m		2.56 m	
16_α_	1.56 m	33.6	1.70 m	33.7	1.72 m	33.4	1.25 ^a^	32.7	1.11 m	33.2	2.07 m	24.1
16_β_	1.96 m		1.96 m		2.02 m		2.30 m		1.98 m		2.15 m	
17		47.3		47.0		47.1		49.0		48.5		47.0
18		133.7		133.8		132.6		132.0		133.6	3.51 m	39.8
19_α_	1.82 m	37.7	1.75 m	37.4	1.77 m	41.2	2.36 m	36.6	2.18 m	41.0	1.50 m	41.2
19_β_	3.29 m		2.65 m		2.53 d (13.5)		3.10 d (14.5)		2.73 d (12.5)		2.12 d (13.5)	
20		45.7		37.5		34.1		37.8		33.2		36.6
21_α_	1.30 m	34.2	1.24 m	32.3	1.25 m	37.4	1.76 m	32.4	1.36 m	37.4	1.29 m	28.7
21_β_	2.06 m		1.60 m		1.40 m		1.93 m		1.72 m		1.73 m	
22_α_	1.34 m	37.9	1.32 m	36.0	1.40 ^a^	36.1	1.63 m	35.2	1.53 m	36.2	1.94 ^a^	31.7
22_β_	2.30 d (12.5)		2.19 m		2.30 m		2.66 d (13.5)		2.66 d (14.0)			
23	1.09 s	26.9	1.07 s	26.9	0.97 s	28.2	1.25 ^a^	28.2	1.34 s	27.6	1.25 s	28.9
24	1.04 s	21.2	1.02 s	21.2	0.77 s	15.5	1.05 s	16.1	1.11 s	15.9	1.07 s	16.5
25	1.04 ^a^	17.9	1.02 ^a^	17.9	0.93 s	18.3	0.90 ^a^	18.2	0.92 s	18.3	1.24 s	25.4
26	0.86 s	17.0	0.82 s	16.8	0.82 s	16.9	1.06 ^a^	16.4	1.06 s	16.8	1.47 s	20.9
27	0.93 s	20.0	0.97 s	20.1	0.98 s	19.8	1.02 ^a^	19.7	1.14 s	20.1	1.22 s	20.4
28		180.2		180.2		176.9		176.0		179.9		176.8
29	1.17 s	29.0	0.90 s	27.3	0.79 s	24.3	1.17 s	27.3	0.92 ^a^	24.4	3.55 br s	73.6
30		181.9	3.18 d (10.0)	67.0	0.91 s	32.5	3.68 d (10.4)	66.4	0.94 ^a^	32.6	1.09 s	19.6
			3.38 d (10.0)				3.76 d (10.4)					
sugar 1					28-*O*-Glc		28-*O*-Glc		3-*O*-Glc		28-*O*-Glc	
1					5.45 d (8.0)	95.9	6.44 d (8.0)	96.6	4.98 d (8.0)	105.3	6.41 d (8.0)	96.0
2					3.27 m	74.1	4.21 m	73.9	4.22 m	84.2	4.25 m	74.2
3					3.37 m	78.6	4.31 m	78.9	3.99 m	78.7	4.29 m	78.9
4					3.30 m	71.3	4.37 m	71.0	4.22^a^	71.8	4.40 m	71.0
5					3.31 m	78.5	4.10 m	79.4	4.37 m	78.4	4.04 m	79.4
6					3.66 m, 3.85 m	62.6	4.41 m, 4.50 m	62.5	4.41 m, 4.61 m	63.0	4.42 m, 4.48 m	62.4
sugar 2									Xyl			
1									5.32 d (7.0)	107.2		
2									4.17 m	76.8		
3									4.20 m	78.7		
4									4.28 m	71.3		
5									3.73 m, 4.43 m	67.8		

^a^ Overlapped with other signals.

## Supplementary Materials

Supplementary materials can be accessed at: http://www.mdpi.com/1420-3049/21/12/1700/s1.

## References

[1] Institute of botany, the Chinese academy of sciences (1978). Flora of China.

[2] Wang Z.Z., Zheng H.C., Su Z.W., Li C.H. (1994). Determination of essential oil, amino acids and trace elements form root cortex of *Aralia dasyphylla* Miq. Acad. J. Sec. Mil. Med. Univ..

[3] Setsuo S., Shigeya S., Norihiro T., Yoichi N., Keishi N., Madoka I., Isao I. (1990). Saponins from the leaves of *Aralia elata* Seem. (Araliaceae). Chem. Pharm. Bull..

[4] Hideaki K., Michiko N., Yukiko T., Masanori K., Akira U., Motoyosh S. (1989). Studies on the udosaponin A, B, C, D, E and F from *Aralia cordata* Thunb. Chem. Pharm. Bull..

[5] Wei L., Hongwei F., Hong B., Tatsunori S., Hiroyoshi K., Kazuo K. (2009). Triterpenoid saponins from *Rubus ellipticus* var. *obcordatus*. J. Nat. Prod..

[6] Linli B., Xiangrong T., Fang D., Liangjiang H., Haifeng T., Siwan W. (2012). New antioxidant and antiglycation active triterpenoid saponins from the root bark of *Aralia taibaiensis*. Fitoterapia.

[7] Xia W., Lei W., Guocai W., Hui W., Yi D., Xinxin Y., Wencai Y., Yaolan L. (2013). Triterpenoid saponins from rhizomes of *Paris polyphylla* var. *yunnanensis*. Carbohydr. Res..

[8] Maria C.M.Y., Adriana P., Edison P.C., Mitsue H., Massao Y., Toshie K. (1997). ^13^C NMR analysis of monodesmosideic saponins from *Gomphrena macrocephala*. Phytochemistry.

[9] Qizhi W., Xiaofeng L., Yu S., Fuqin G., Yu C., Xiangyun W., Ming W., Xu F. (2012). Two new nortriterpenoid saponins from *Salicornia bigelovii* Torr. and their cytotoxic activity. Fitoterapia.

[10] Yoshiyasu F., Hiroyuki M., Hiromi F., Miyako T. (2002). Triterpenoids from *Viburnum suspensum*. Phytochemistry.

[11] Ilias M., Khalid A.E.S., Jaber S.M., Mansour S.A., Farouk S.F., Alice M.C., Charles D.H., Stephen O., Alejandro M.S.M. (2000). Bioactive 12-oleanene triterpene and secotriterpene acids from *Maytenus undata*. J. Nat. Prod..

[12] Chunmei L., Hexiang W., Shilei W., Kun G. (2008). Oleanane-type triterpenes from the flowers and roots of *Saussurea muliensis*. J. Nat. Prod..

[13] Hui M., Youngyan S., Yunfei Y., Huanhuan Z., Jiao W., Weiyun Z., Lijuan Z. (2016). Herbicidal and cytotoxic constituents from *Aralia armata* (WALL.) SEEM. Chem. Biodivers..

[14] Perveen S., Taweel A.M., Fawzy G.A., Ibrahim T.A., Malik A., Khan A. (2014). Cholinesterase inhibitory triterpenes from *Perovskia atriplicifolia*. Asian J. Chem..

