# Supplementary material for: Six New Triterpene Derivatives from Aralia chinensis Var. dasyphylloides
Source: Molecules. 2016 Dec 9;21(12):1700. doi: 10.3390/molecules21121700 (PMC6273084; doi:10.3390/molecules21121700)
Supplement: Supplementary file 1 [file molecules-21-01700-s001.pdf]

Supplementary Materials: Six New Triterpene Derivatives from *Aralia chinensis* Var. *dasyphylloides*

Ruixi Gao, Maochuan Liao, Xianju Huang, Yu Chen, Guangzhong Yang and Jun Li

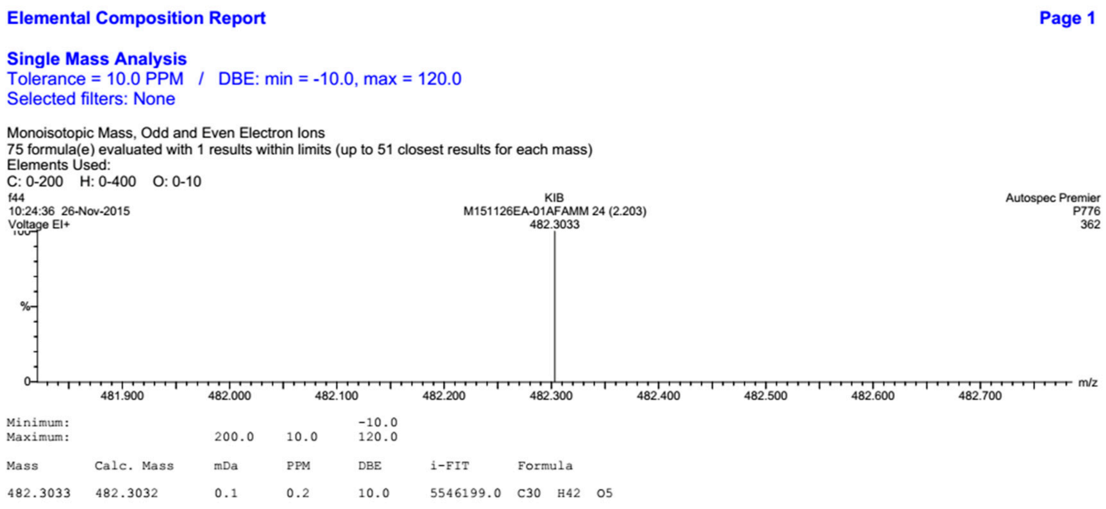
Elemental Composition Report Page 1

Single Mass Analysis
Tolerance = 10.0 PPM / DBE: min = -10.0, max = 120.0
Selected filters: None

Monoisotopic Mass, Odd and Even Electron Ions
75 formula(e) evaluated with 1 results within limits (up to 51 closest results for each mass)
Elements Used:
C: 0-200 H: 0-400 O: 0-10

Minimum:				-10.0		
Maximum:		200.0	10.0	120.0		
Mass	Calc. Mass	mDa	PPM	DBE	i-FIT	Formula
482.3033	482.3032	0.1	0.2	10.0	5546199.0	C30 H42 O5

Figure S1. HR-EI-MS data of compound 1.

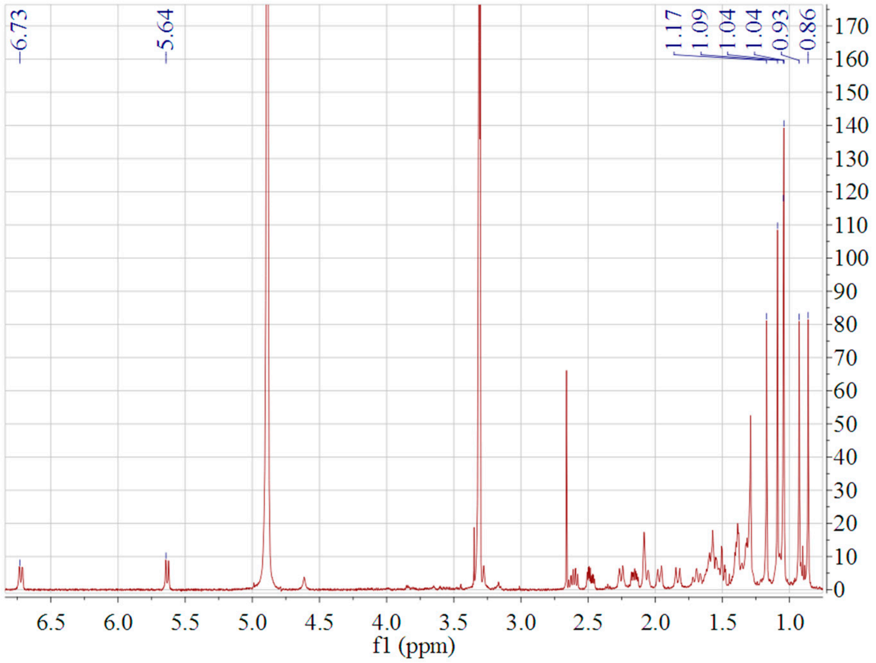

Figure S2. ^{1}H-NMR data of compound 1 (500 MHz, CD_3OD).

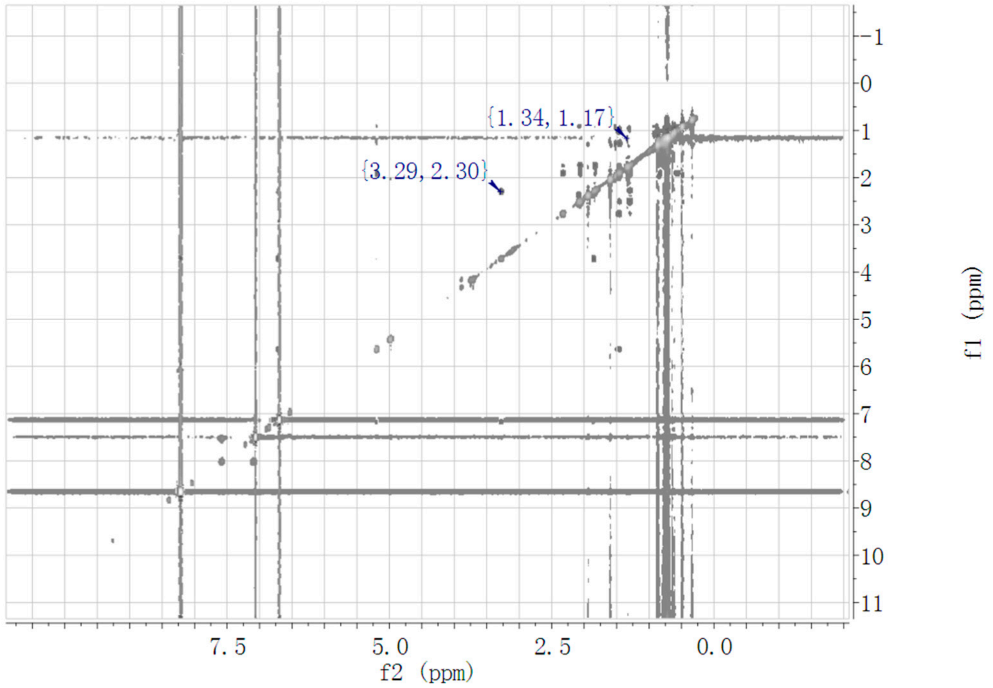

Figure S3. NOESY data of compound 1.

Qualitative Analysis Report

**Data Filename**	140514ESINA2.d	**Sample Name**	f45
**Sample Type**	Sample	**Position**	
**Instrument Name**	Agilent G6230 TOF MS	**User Name**	KIB
**Acq Method**	ESIN.m	**Acquired Time**	5/14/2014 5:29:55 PM
**IRM Calibration Status**	Success	**DA Method**	ESIN.m
**Comment**			

**Sample Group**		**Info.**
**Acquisition SW**	6200 series TOF/6500 series	
**Version**	Q-TOF B.05.01 (B5125.1)	

User Spectra

Fragmentor Voltage	Collision Energy	Ionization Mode
200	0	ESI

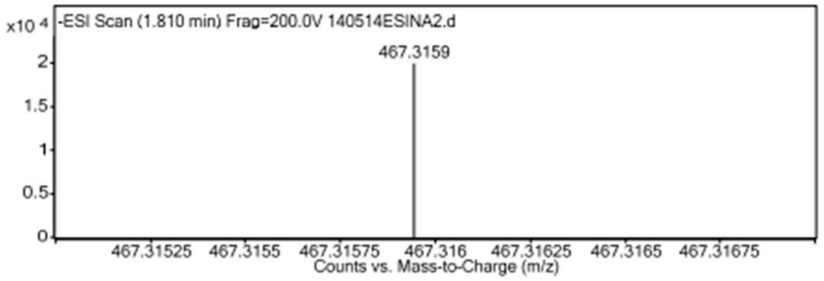

Peak List

m/z	z	Abund
423.3268	1	141367.11

Formula Calculator Element Limits

Element	Min	Max
C	0	200
H	0	400
O	2	20

Formula Calculator Results

Formula	CalculatedMass	Mz	Diff.(mDa)	Diff. (ppm)	DBE
C30 H43 O4	467.3161	467.3159	0.2	0.4	9.5

--- End Of Report ---

Figure S4. HR-ESI-MS data of compound 2.

Qualitative Analysis Report

**Data Filename**	140514ESINA3.d	**Sample Name**	f47
**Sample Type**	Sample	**Position**	
**Instrument Name**	Agilent G6230 TOF MS	**User Name**	KIB
**Acq Method**	ESIN.m	**Acquired Time**	5/14/2014 5:32:23 PM
**IRM Calibration Status**	Success	**DA Method**	ESIN.m
**Comment**			

**Sample Group**		**Info.**
**Acquisition SW**	6200 series TOF/6500 series	
**Version**	Q-TOF B.05.01 (B5125.1)	

User Spectra

Fragmentor Voltage	Collision Energy	Ionization Mode
200	0	ESI

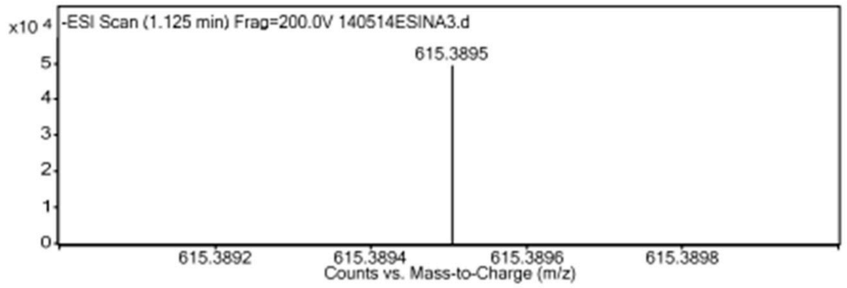

Peak List

m/z	z	Abund
729.3835	1	285384.22

Formula Calculator Element Limits

Element	Min	Max
C	0	200
H	0	400
O	2	20

Formula Calculator Results

Formula	CalculatedMass	Mz	Diff.(mDa)	Diff. (ppm)	DBE
C36 H55 O8	615.3897	615.3895	0.2	0.3	9.5

--- End Of Report ---

Figure S5. HR-ESI-MS data of compound 3.

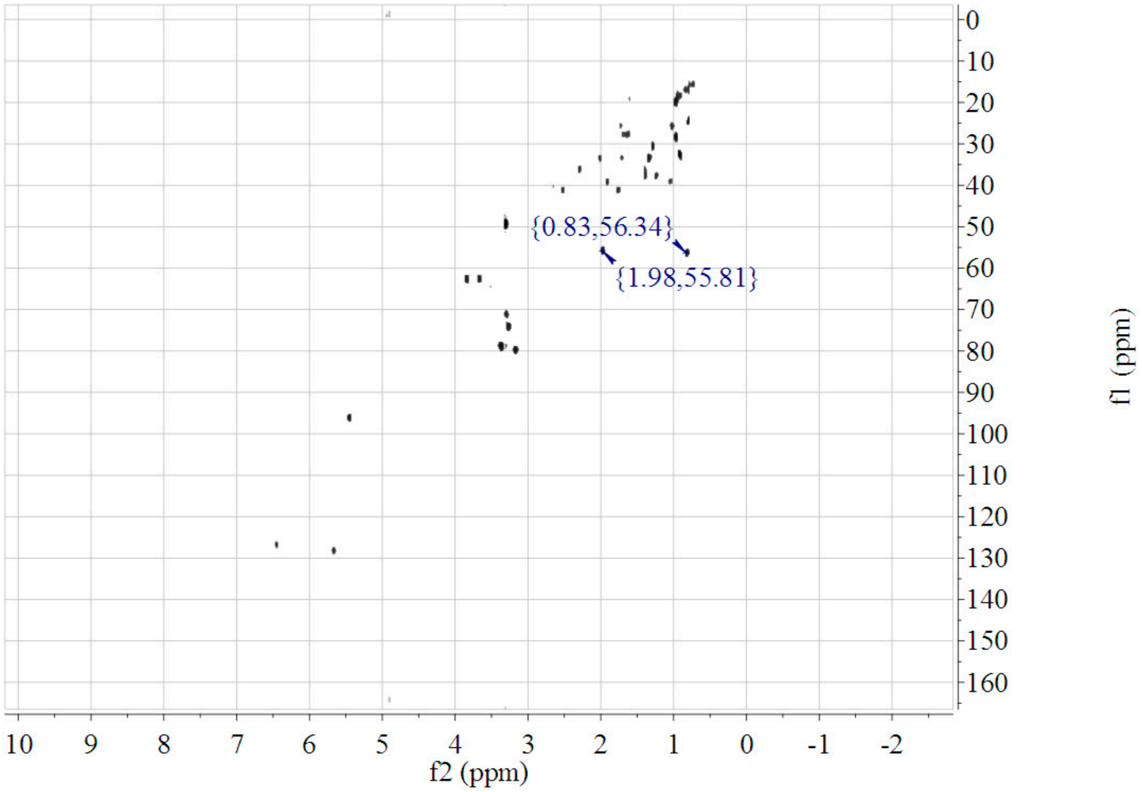

Figure S6. HSQC data of compound 3.

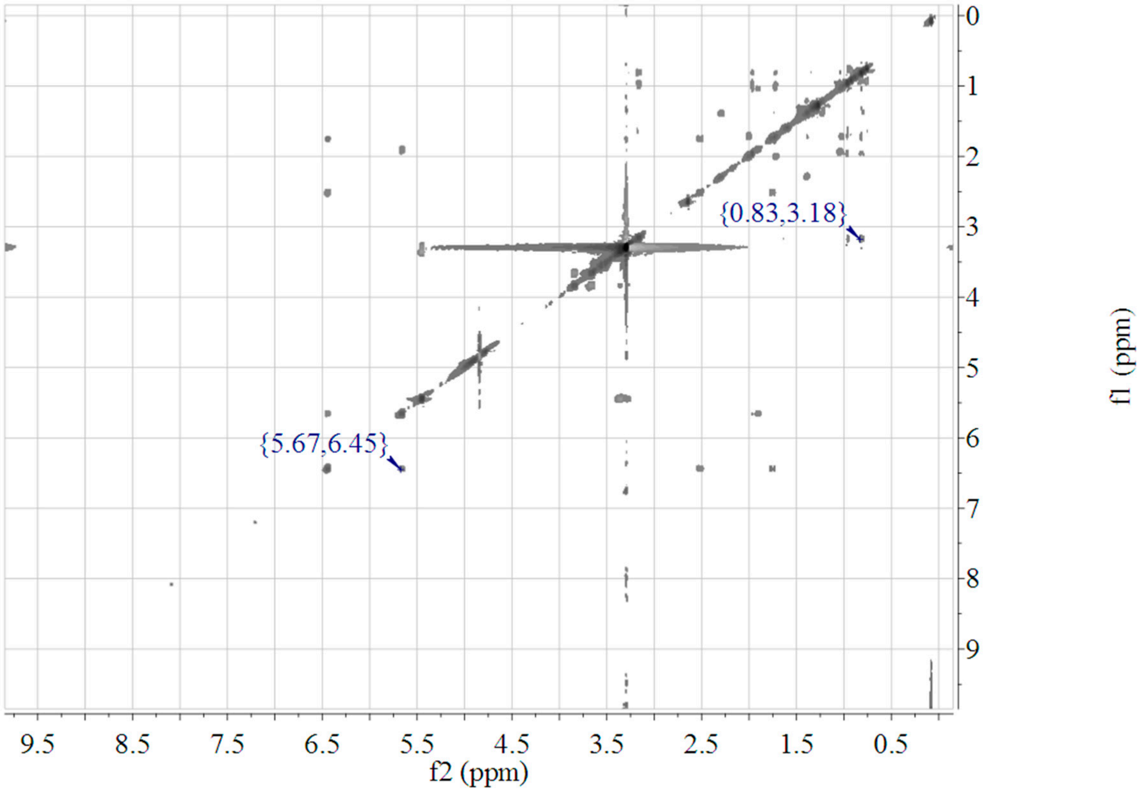

Figure S7. NOESY data of compound 3

Qualitative Analysis Report

**Data Filename**	140504ESINA1.d	**Sample Name**	CP11
**Sample Type**	Sample	**Position**	
**Instrument Name**	Agilent G6230 TOF MS	**User Name**	KIB
**Acq Method**	ESIN.m	**Acquired Time**	5/4/2014 9:48:08 AM
**IRM Calibration Status**	Success	**DA Method**	ESIN.m
**Comment**			

Sample Group **Info.**
Acquisition SW 6200 series TOF/6500 series
Version Q-TOF B.05.01 (B5125.1)

User Spectra

Fragmentor Voltage	Collision Energy	Ionization Mode
200	0	ESI

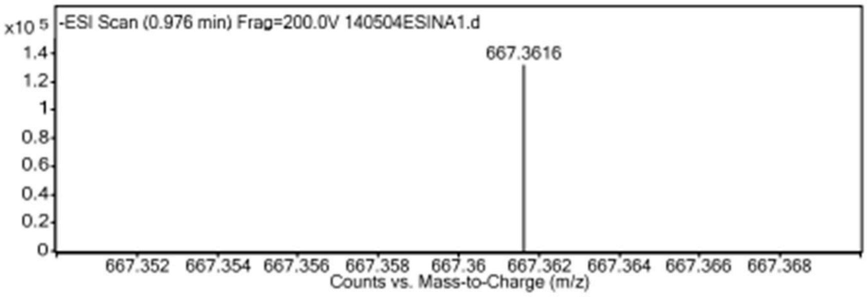

Peak List

m/z	z	Abund	Formula	Ion
667.3616	1	131533.86	C36 H56 Cl O9	M-

Formula Calculator Element Limits

Element	Min	Max
C	0	200
H	0	400
O	2	20
Cl	0	1

Formula Calculator Results

Formula	CalculatedMass	Mz	Diff.(mDa)	Diff. (ppm)	DBE
C36 H56 Cl O9	667.3613	667.3616	-0.3	0.4	8.5

--- End Of Report ---

Figure S8. HR-ESI-MS data of compound 4.

Qualitative Analysis Report

**Data Filename**	140505ESINA2.d	**Sample Name**	CP18
**Sample Type**	Sample	**Position**	
**Instrument Name**	Agilent G6230 TOF MS	**User Name**	KIB
**Acq Method**	ESIN.m	**Acquired Time**	5/5/2014 9:44:20 AM
**IRM Calibration Status**	Success	**DA Method**	ESIN.m
**Comment**			

Sample Group **Info.**

Acquisition SW 6200 series TOF/6500 series

Version Q-TOF B.05.01 (B5125.1)

User Spectra

Fragmentor Voltage	Collision Energy	Ionization Mode
200	0	ESI

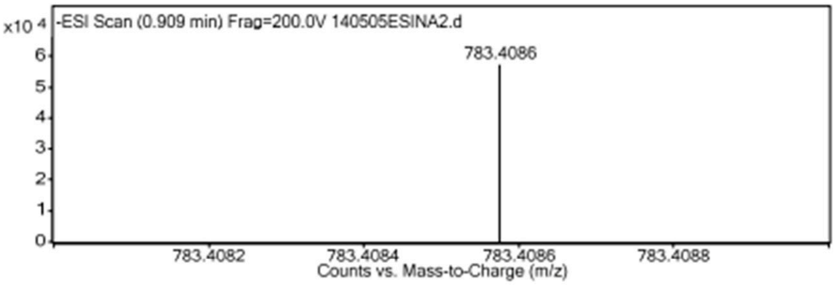

Peak List

m/z	z	Abund
1033.9881	1	66713.21

Formula Calculator Element Limits

Element	Min	Max
C	0	200
H	0	400
O	5	25
Cl	1	1

Formula Calculator Results

Formula	CalculatedMass	Mz	Diff.(mDa)	Diff. (ppm)	DBE
C41 H64 Cl O12	783.4086	783.4086	0.0	0.0	9.5

--- End Of Report ---

Figure S9. HR-ESI-MS data of compound 5.

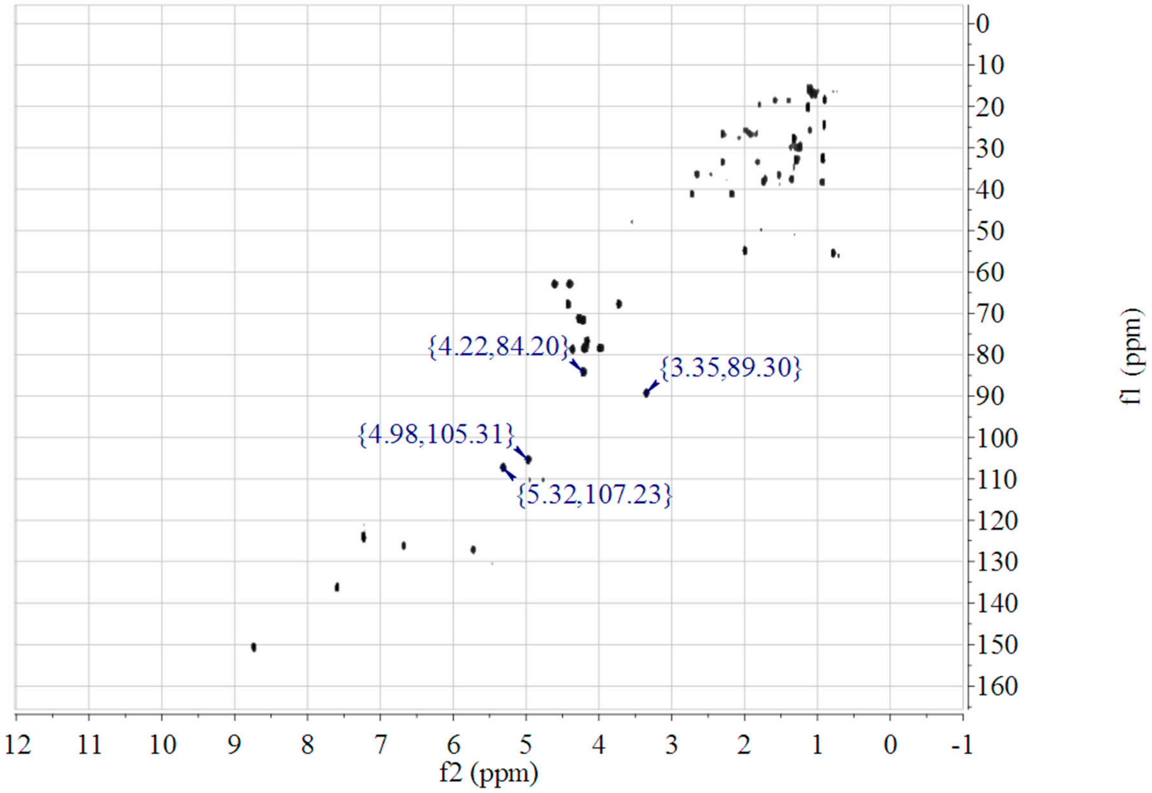

Figure S10. HSQC data of compound 5.

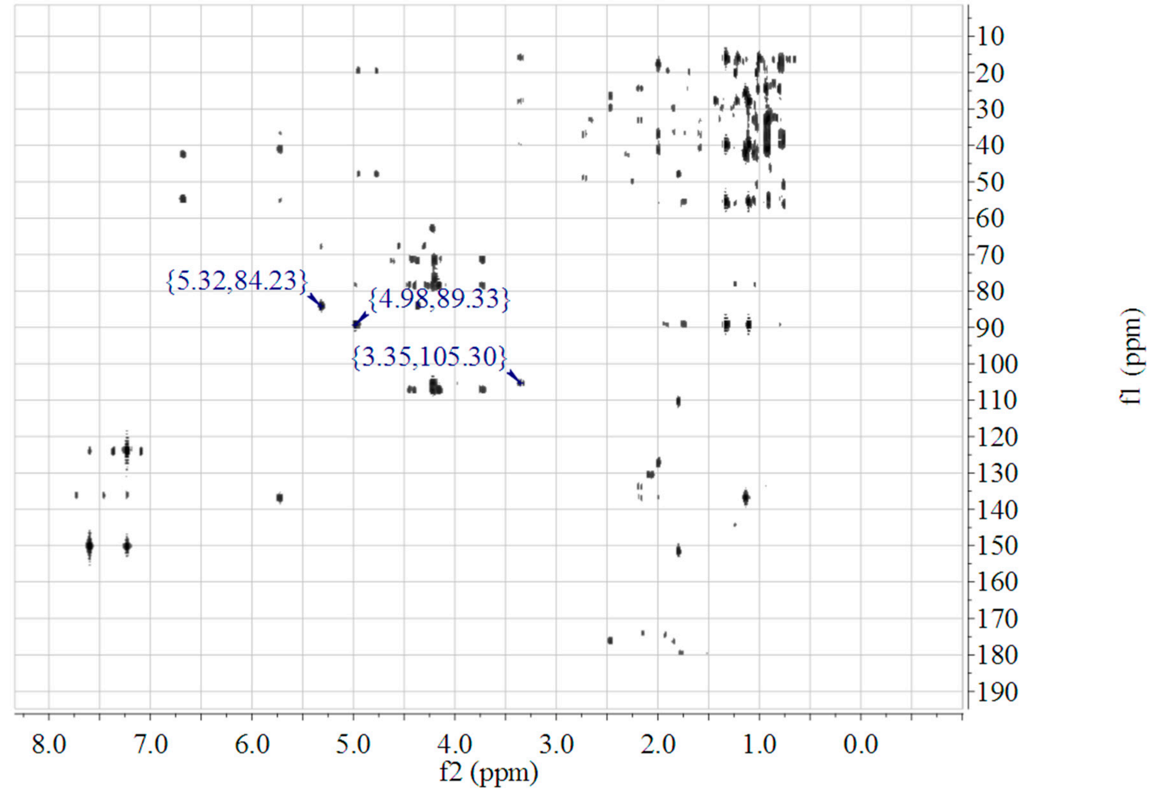

Figure S11. HMBC data of compound 5.

Qualitative Analysis Report

**Data Filename**	140505ESINA1.d	**Sample Name**	CP10
**Sample Type**	Sample	**Position**	
**Instrument Name**	Agilent G6230 TOF MS	**User Name**	KIB
**Acq Method**	ESIN.m	**Acquired Time**	5/5/2014 9:37:19 AM
**IRM Calibration Status**	Success	**DA Method**	ESIN.m
**Comment**			

**Sample Group**		**Info.**
**Acquisition SW**	6200 series TOF/6500 series	
**Version**	Q-TOF B.05.01 (B5125.1)	

User Spectra

Fragmentor Voltage	Collision Energy	Ionization Mode
200	0	ESI

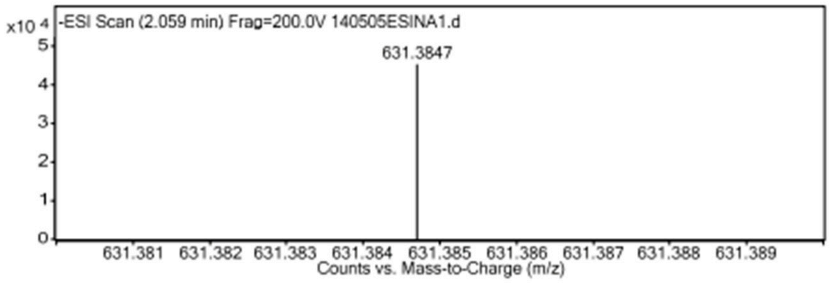

Peak List

m/z	z	Abund
745.378	1	81054.69

Formula Calculator Element Limits

Element	Min	Max
C	0	200
H	0	400
O	3	20

Formula Calculator Results

Formula	CalculatedMass	Mz	Diff.(mDa)	Diff. (ppm)	DBE
C36 H55 O9	631.3846	631.3847	-0.1	0.2	9.5

--- End Of Report ---

Figure S12. HR-ESI-MS data of compound 6.

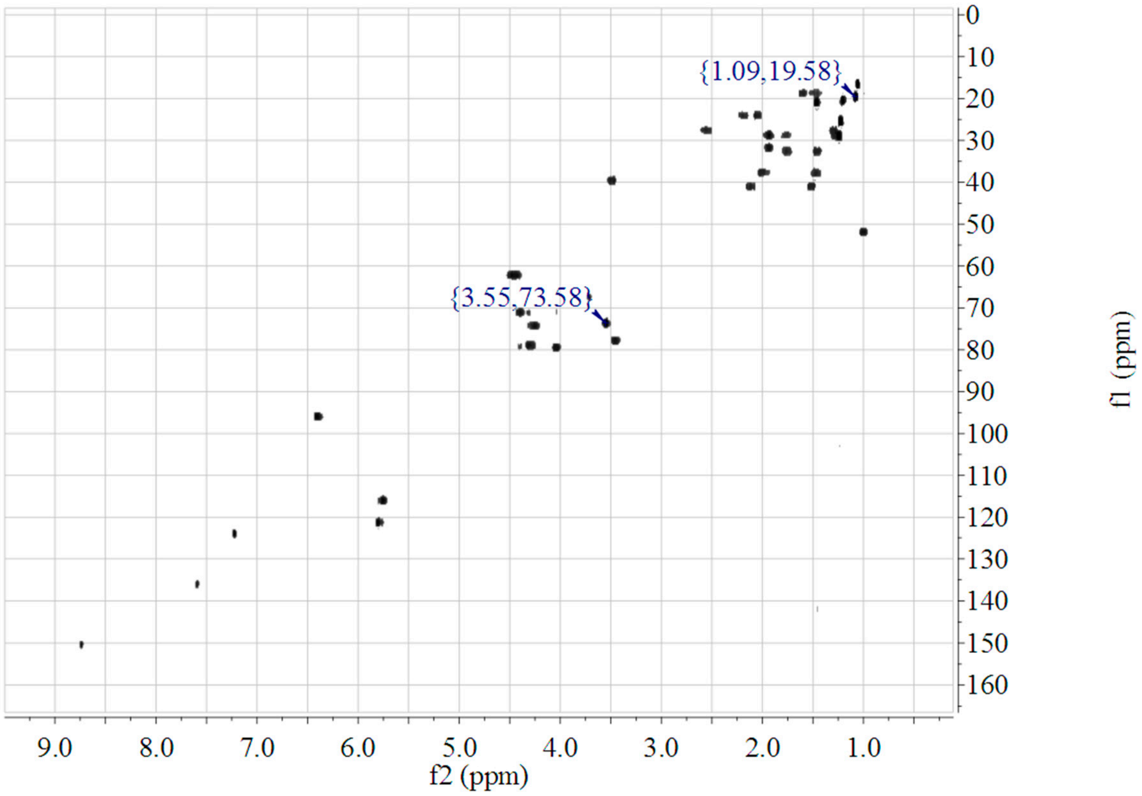

Figure S13. HSQC data of compound 6.

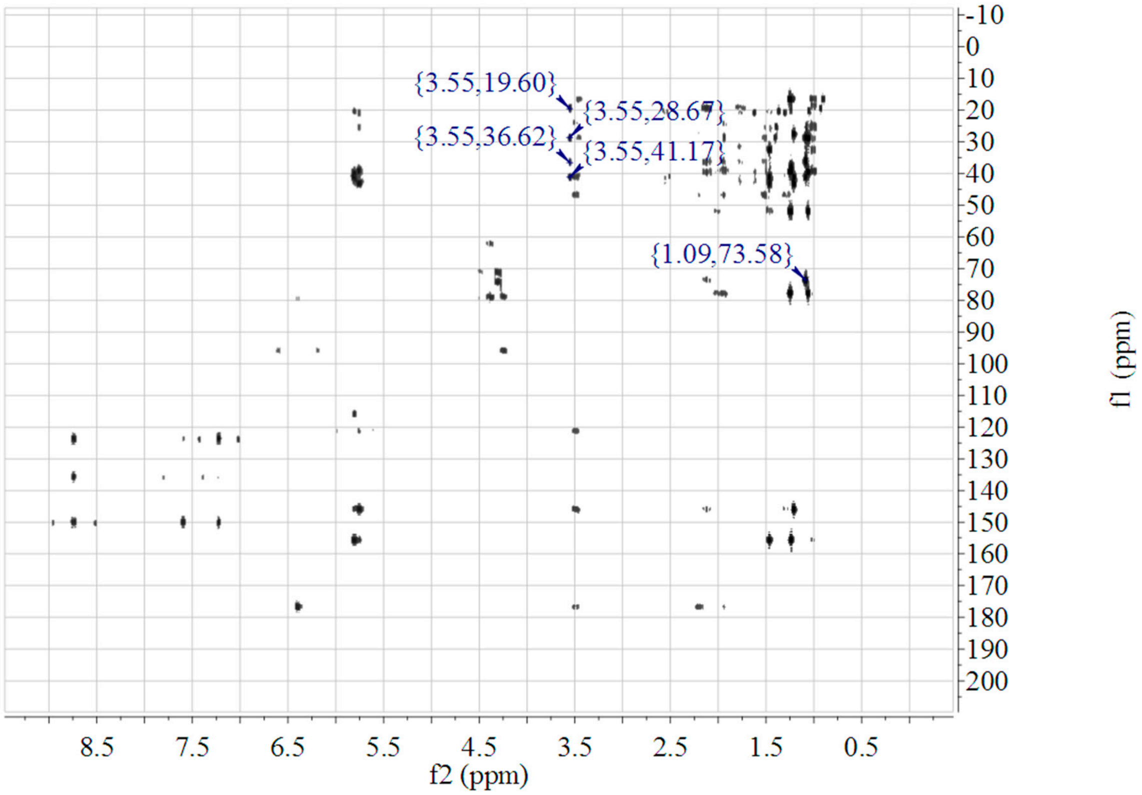

Figure S14. HMBC data of compound 6.

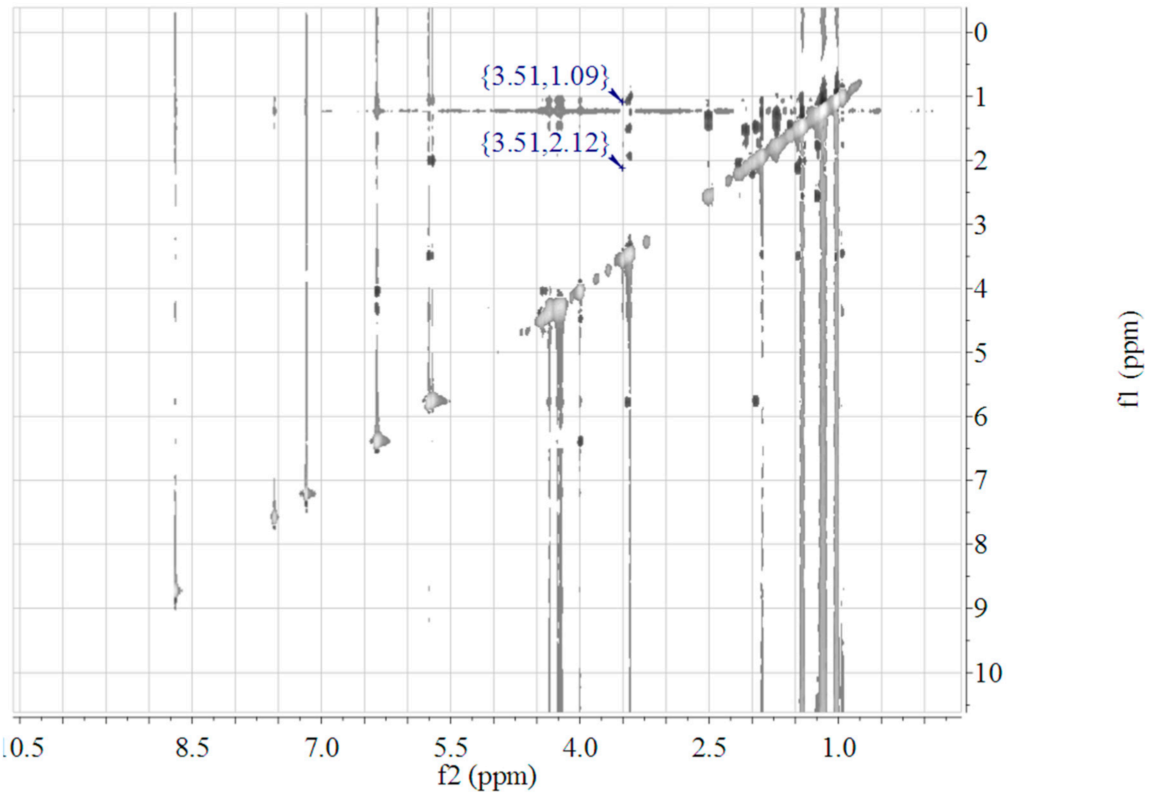

Figure S15. NOESY data of compound 6.

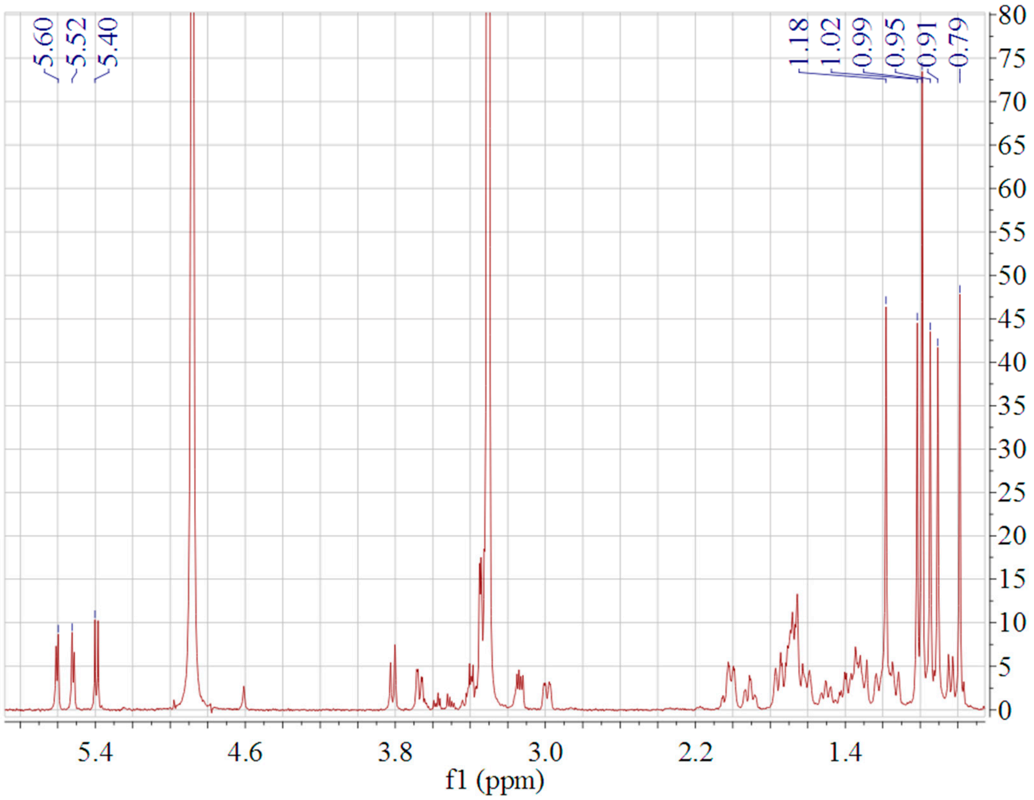

Figure S16. ^{1}H NMR data of compound 7 (500 MHz CD_3OD).

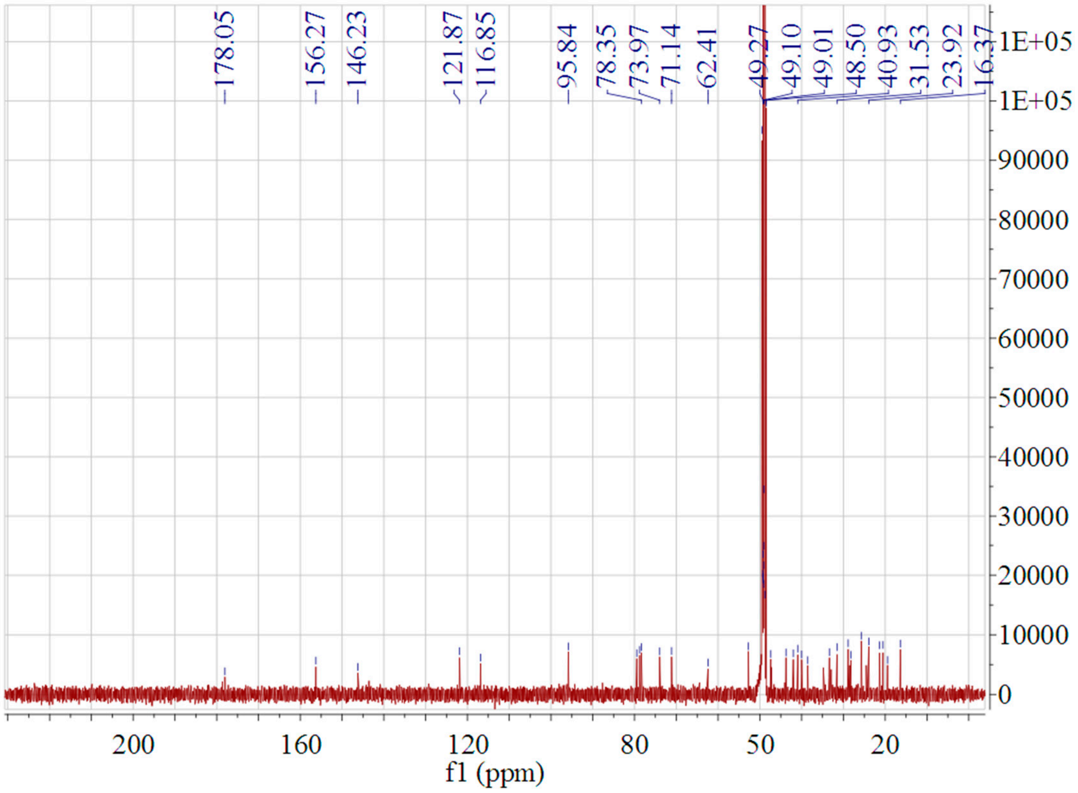

Figure S17. ^{13}C NMR data of compound 7 (125 MHz CD_3OD).

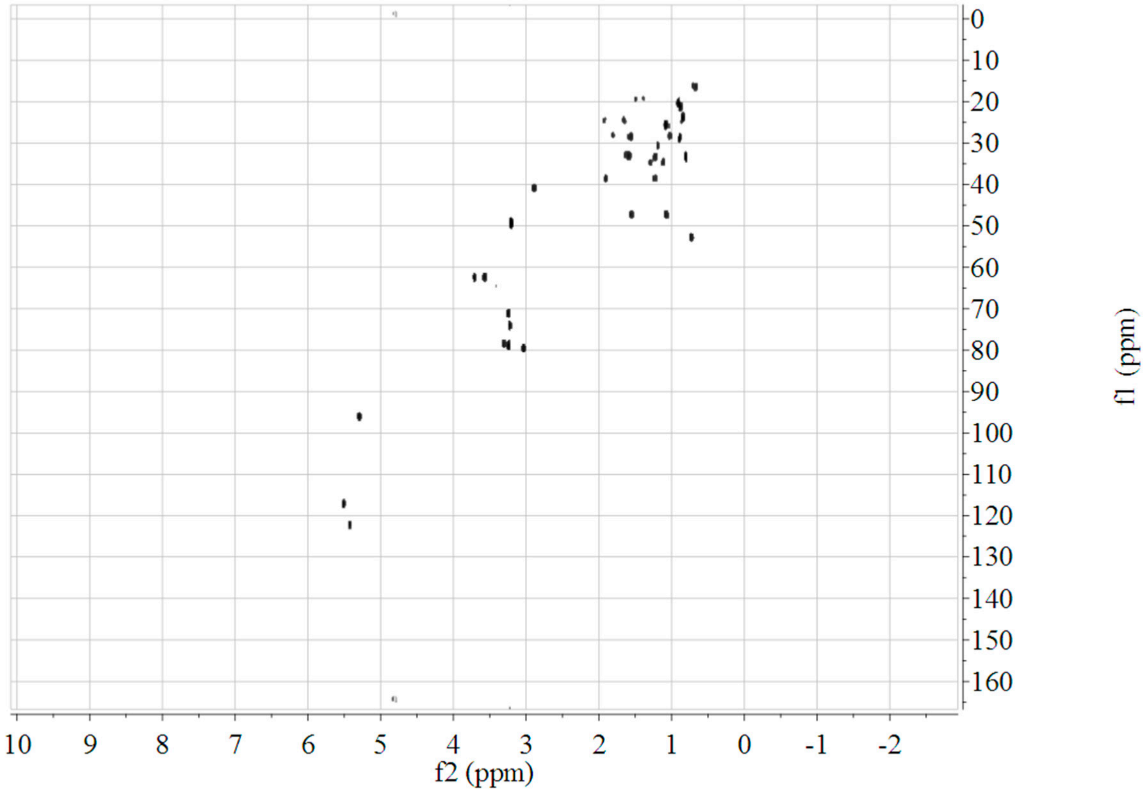

Figure S18. HSQC data of compound 7.

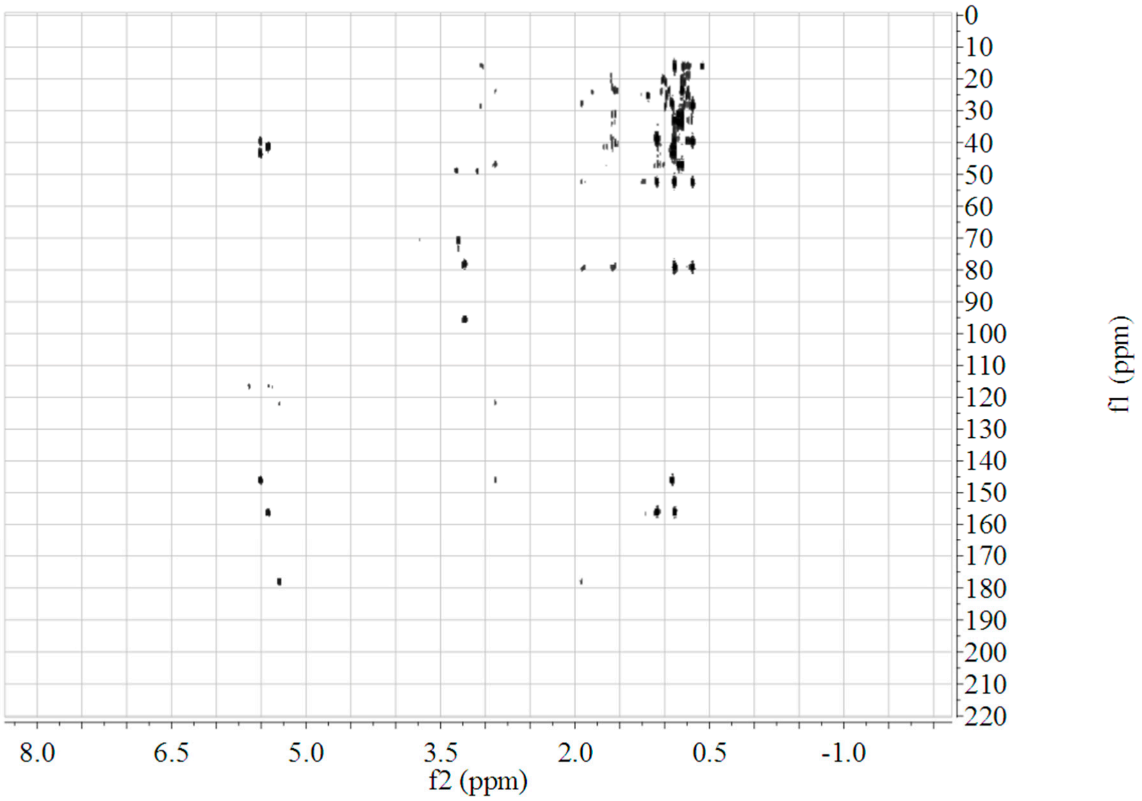

Figure S19. HMBC data of compound 7.

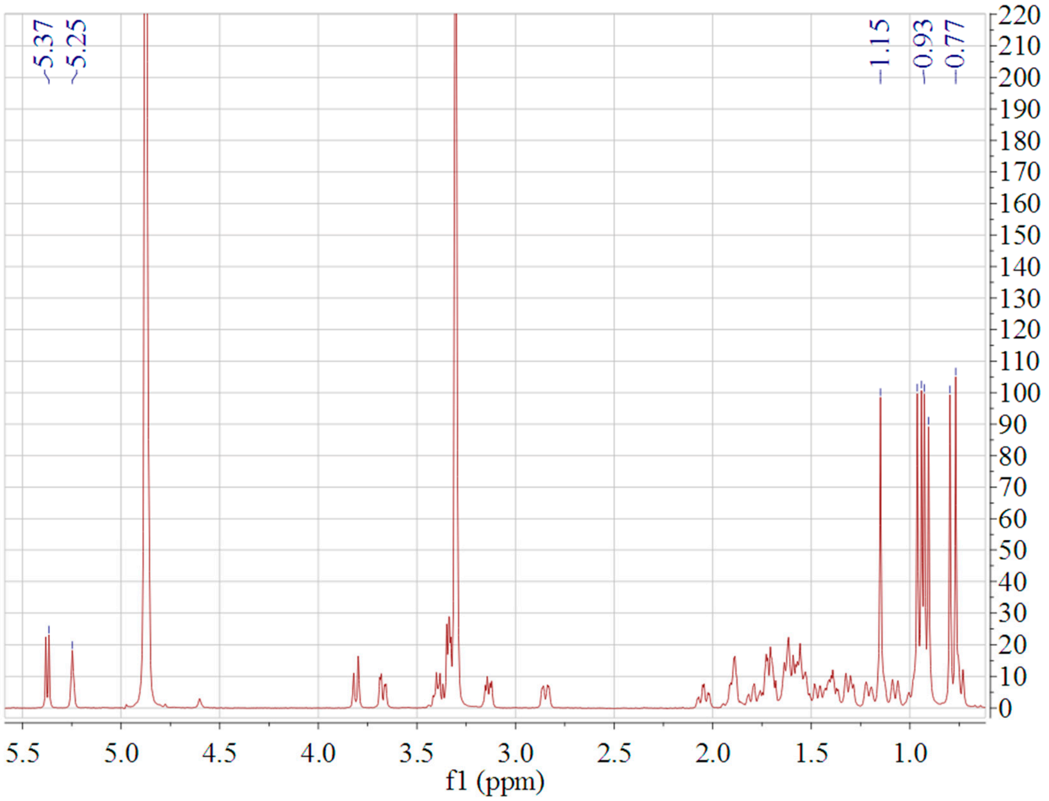

Figure S20. ^1H NMR data of compound 8 (500 MHz CD_3OD).

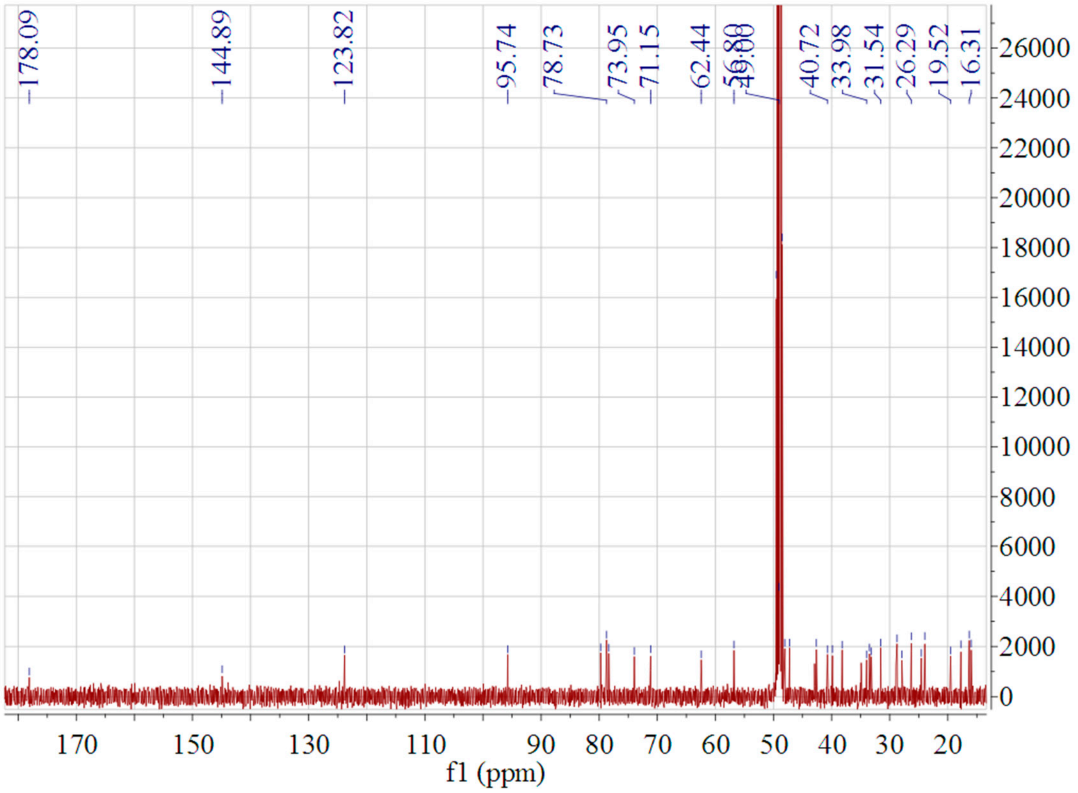

Figure S21. ^{13}C NMR data of compound 8 (125 MHz CD_3OD).

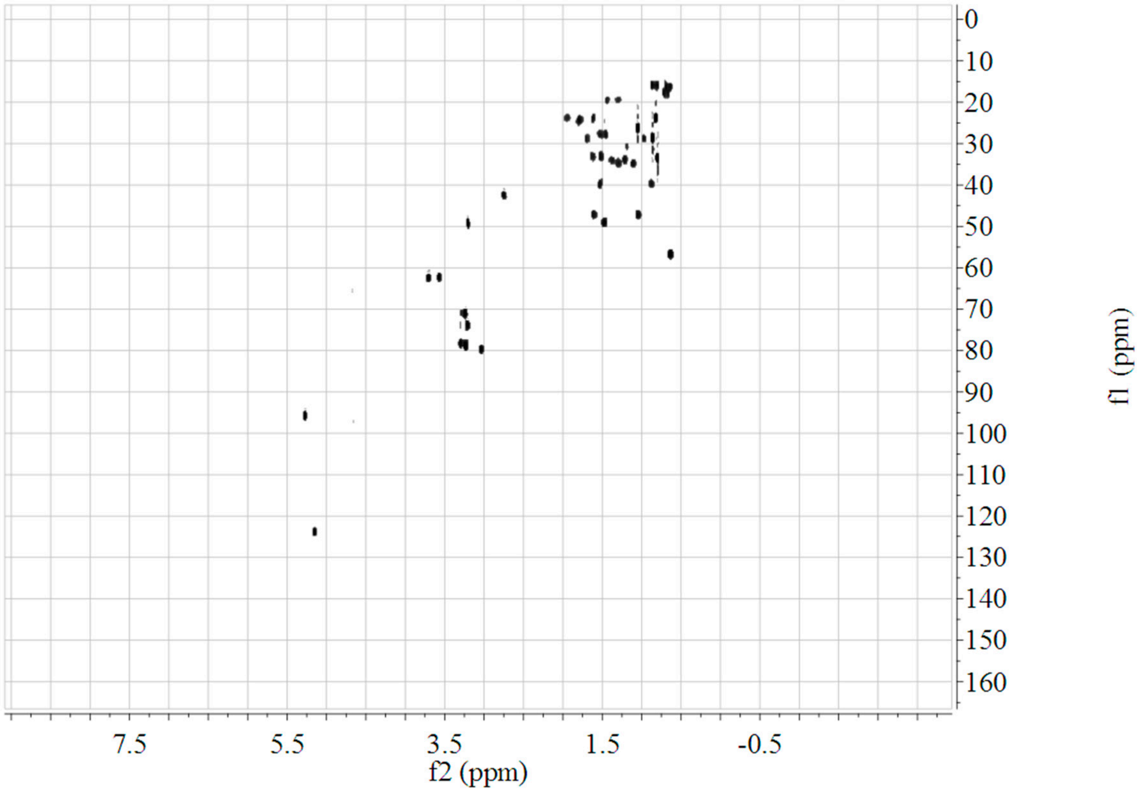

Figure S22. HSQC data of compound 7.

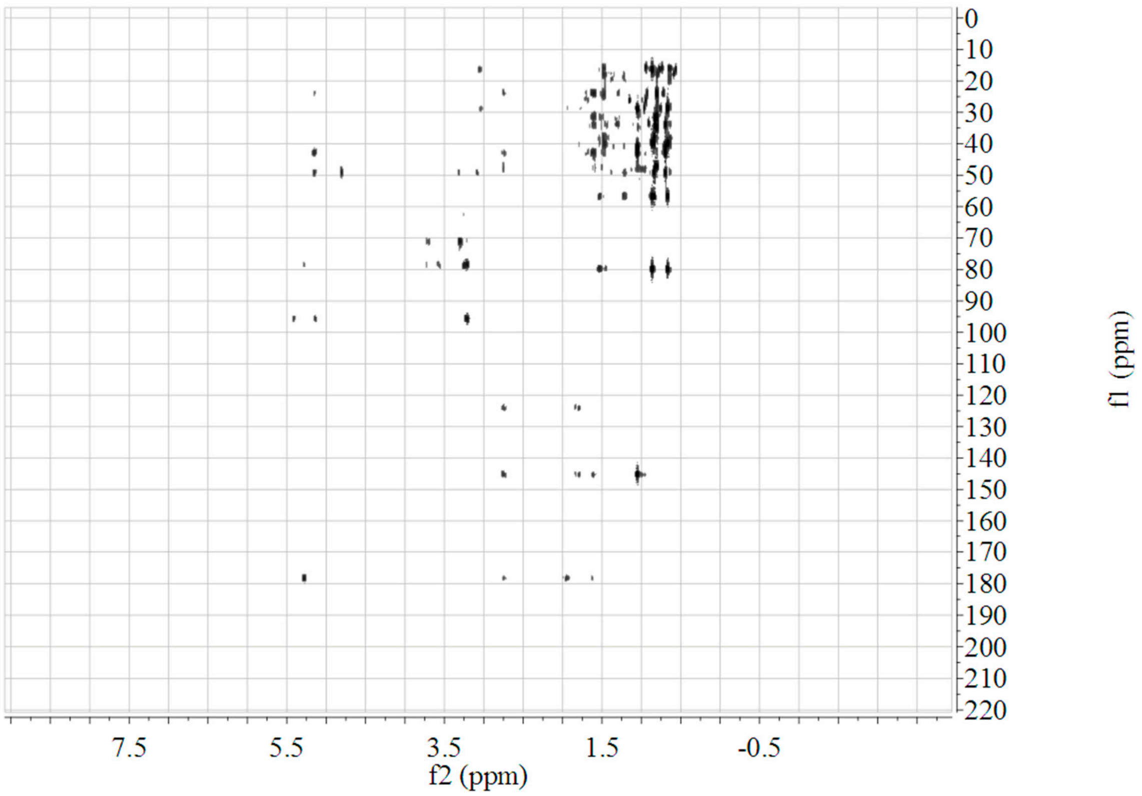

Figure S23. HMBC data of compound 8.
